# Regional Comparison in Cerebral Performance Outcome of Out-of-Hospital Cardiac Arrest: The All-Japan Utstein Registry

**DOI:** 10.7759/cureus.72622

**Published:** 2024-10-29

**Authors:** Ryuhei Igeta, Shunichi Otaka, Takahiro Imaizumi, Kentaro Kajino, Danya Khoujah, Fumihito Ito, Takuro Endo, Takuyo Chiba, Shunya Ikeda, Takashi Shiga

**Affiliations:** 1 Emergency Medicine, International University of Health and Welfare, Narita Hospital, Narita, JPN; 2 Public Health, Graduate School of Medicine, International University of Health and Welfare, Narita, JPN; 3 Advanced Medicine, Nagoya University Hospital, Nagoya, JPN; 4 Emergency Medicine, Kansai Medical University, Osaka, JPN; 5 Emergency Medicine, University of Maryland School of Medicine, Maryland, USA; 6 Emergency Medicine, AdventHealth Tampa, Florida, USA

**Keywords:** cardiac arrest outcome, cerebral performance category, emergency medical service, neurological outcomes, out of hospital cardiac arrest, regional differences, resuscitation outcomes

## Abstract

Background: Past studies have reported regional differences in the prognosis of out-of-hospital cardiopulmonary arrest (OHCA) in Japan, but there is a lack of sufficient information to explain the changes in neurological prognosis over time.

Methods: Using the All-Japan Utstein Registry, a nationwide population-based OHCA database, we included non-traumatic OHCA patients resuscitated by emergency responders across all seven regions from January 1, 2011, to December 31, 2017. The primary endpoint was a favorable neurological outcome one month after cardiac arrest, compared among these seven regions. The secondary endpoint was one-month survival after OHCA.

Results: There were 880,043 OHCA patients, of whom 721,455 (82.0%) were included in the analysis. Overall, a total of 17,685 (2.45%) patients survived with favorable neurological outcomes. Unadjusted neurological favorable outcomes varied regionally, ranging from 1.64% to 3.37% (rate difference: 1.73%; 95%CI: 1.57-1.89%). The East and Northeast had a significantly lower rate compared to the Midwest region (unadjusted rate ratio: 0.73; 95%CI: 0.70-0.76; p<0.001, 0.63; 95%CI: 0.59-0.67; p<0.001, respectively). The significant difference remained after adjustment for patient factors and prehospital factors (adjusted rate ratio: 0.68; 95%CI: 0.64-0.72, p<0.01, 0.70; 95%CI: 0.67-0.73, p<0.01, respectively). The secondary outcome, survival at one month, also showed similar regional differences.

Conclusion: In this post-hoc analysis, nationwide, population-based study in Japan, we found that regional differences in the neurologic prognosis of OHCA patients persist. The difference has narrowed from previous years, but the causes still need to be investigated and improved in the future.

## Introduction

Cardiac arrest is the cessation of cardiac mechanical activity, as confirmed by the absence of signs of circulation [1]. Out-of-hospital cardiac arrest (OHCA) is a major public health problem worldwide, annually affecting more than 350,000 individuals (110.8 individuals per 100,000 population) in the United States between 2005 and 2015 [2], 67-170 per 100,000 individuals in Europe [3], and more than 125,000 individuals in Japan [4]. The incidence rate has increased by more than 10,000 people from 2009 to 2018 [4].

Regional differences in neurological outcomes after OHCA have been reported in various countries (such as the United States, Europe, Denmark, and Turkey) [5-8]. In the United States, there was marked variation in rates of survival to discharge (range: 3.4%-22.0%; median odds ratio: 1.40; 95% CI: 1.32-1.46) and survival with functional recovery (range: 0.8%-21.0%; median odds ratio: 1.53; 95%CI: 1.43-1.62) [5]. These differences were due to the different responses of witnesses in local communities. In a study of 28 European countries, 8% of OHCA patients survived hospital discharge(range: 0%-18%) [6]. Differences in bystander resuscitation, the effectiveness of emergency medical systems, hospital treatment, culture, and attitudes towards cardiopulmonary resuscitation may be factors in the large variations seen in different countries. Reports from Denmark showed a 30-day survival rate of 12.0% (range: 8.5%-13.8%), and that there were regional differences even within a single country [7]. In Japan, the neurologically favorable survival rate was 2.4% (range: 1.9%-3.1%), and the one-month survival rate was 4.9% (range: 3.7%-6.1%) [9]. Adjustment for patient- and prehospital-level covariates accentuated the disparity (adjusted rate ratio: 0.52; 95%CI: 0.51-0.54; p<0.001). The cause of these regional differences in Japan is not clear, given the universal health care and relatively uniform prehospital and medical care throughout the country [10]. A Japanese study reported that the number of basic life support (BLS) providers and public access automated external defibrillators (AED) did not explain regional differences in the neurological prognosis of OHCA [11]. Other Japanese studies using data from 2005 to 2014 showed that the prognosis of OHCA tended to improve over time [12]. Japan has universal health insurance, and access to hospitals is good regardless of living standards, but the reason for the regional differences in the prognosis of OHCA is not known.

The purpose of this study is to analyze regional differences in the neurological outcomes of OHCA in Japan based on the Utstein database from 2011 to 2017, as well as the one-month survival following OHCA.

## Materials and methods

Study design and participants

The All-Japan Utstein registry was collected by the Fire and Disaster Management Agency in 2005. It is a prospective, nationwide, population-based registry system of all OHCAs for whom resuscitation was attempted by emergency medical service (EMS) and subsequently transported to the hospital.

We included adults (20 years) with EMS-treated OHCA of medical origin from January 1, 2011, to December 31, 2017, within the registry. We excluded patients with missing data, external causes of cardiac arrest (such as trauma, toxins, drowning, or anaphylaxis), and those with extremely long prehospital time (time from call to initiation of CPR by EMS at the scene 60 minutes, time from initiation of CPR by EMS at the scene to hospital arrival 60 minutes, or time from call to hospital arrival 120 minutes), as previously defined [13].

The institutional review board of the International University of Health and Welfare Narita Hospital approved the study with a waiver of informed consent (approved number: 21-Im-039).

Study setting and EMS system in Japan

Japan had a population of approximately 127 million in 2014 [14]. As per the Ministry of Health, Labor, and Welfare, at least one emergency medical center should be available per one million residents, and 284 centers were established by 2017 [15]. All EMS performed cardiopulmonary resuscitation (CPR) according to the Japanese CPR guidelines, which are based on the International Liaison Committee on Resuscitation recommendations [16]. EMS in Japan are not allowed to terminate resuscitation out of the hospital, except for specific situations (e.g., decapitation, incineration, decomposition, rigor mortis, or dependent cyanosis). Therefore, the majority of EMS-treated OHCA victims were transferred to hospitals and included in the registry.

The selection of the hospital for transportation is at the discretion of the EMS, although some regions have their own regulations, such as prioritizing transportation to a hospital where the patient previously received their care.

There are usually three EMS personnel in an ambulance, and almost all ambulances are staffed by at least one emergency life-saving technician (ELST). ELSTs may establish intravenous access, perform advanced airway management, and administer adrenaline en route to the hospital, all under the direction of the designated medical officer. EMS personnel may perform usual BLS such as chest compressions, bag-valve-mask ventilation, and defibrillation without requiring a physician’s order.

Data collection and quality control

Data were collected prospectively with an Utstein-style form, including the following: age, sex, date of cardiac arrest, etiology of cardiac arrest, first documented cardiac rhythm, presence and type of CPR by a bystander (compression-only vs conventional), use of a public-access AED, presence of an ELST in the ambulance, administration of adrenaline, advanced airway management, and return of spontaneous circulation (ROSC) prior to hospital arrival. The age groups were classified into four categories (<20, 20-64, 65-79, >79) [17]. A series of EMS times were recorded, such as time of initial call, time of EMS dispatch, ambulance arrival at the scene, initial patient contact, initiation of CPR, and hospital arrival. The etiologies of cardiac arrest were recorded in the registry as cardiogenic (definite or presumptive), cerebrovascular, respiratory, malignant, external causes (trauma, toxin, drowning, and anaphylaxis), hypothermia, and others. Attribution of cardiac or non-cardiac etiology was made by the attending physicians in the emergency department in collaboration with the EMS personnel. Furthermore, the EMS personnel queried the medical control director at the hospital one month after the OHCA event to confirm the etiology of the arrest. If there was a disagreement on the etiology, the determination at one month was prioritized. Outcome data included neurological status and survival one month after the event, which were obtained by the treating EMS personnel by querying the medical director at the receiving hospital. To collect one-month follow-up data, the EMS personnel who treated each patient with OHCA queried the medical control director at the hospital.

The annual incidence of OHCA was calculated per 100,000. The incidence in persons of any age was adjusted for age categories and sex data from the 2015 census for Japan. We defined seven geographic regions (North, Northeast, East, Central, Midwest, West, and South) based on the Ministry of Health, Labour, and Welfare of Japan(Supplemental Appendix) [18]. The Midwest region served as the reference, as per prior literature [9].

Study endpoints

The primary endpoint was a favorable neurological outcome one month after cardiac arrest, using the Glasgow-Pittsburgh Cerebral Performance Category (CPC) scale (category 1, good cerebral performance; 2, moderate cerebral disability; 3, severe cerebral disability; 4, coma or vegetative state; 5, death/brain death)[19]. A favorable neurological outcome was defined as a Glasgow-Pittsburgh CPC of 1 or 2 [9,20]. The secondary endpoint was one-month survival after OHCA.

Statistical analysis

First, we calculated the unadjusted rate of one-month survival with favorable neurological outcomes after OHCA in each geographic region. Second, we used a multivariable Poisson regression model with robust variance adjusting for a priori defined patient-level variables, such as age category, sex, etiology of arrest, and EMS-related covariates including type of bystander CPR, the use of a public-access AED, adrenaline administration, advanced airway device placement, presence of an ELST in ambulance, time intervals (from receipt of initial call to initiation of CPR by EMS, and from initiation of CPR by EMS to hospital arrival). Time interval data were categorized into quartiles and analyzed.

All statistical analyses were performed with STATA, version 16.1 (StataCorp, College Station, TX). All tests were two-tailed, and p<0.05 was regarded as statistically significant.

## Results

Patient and prehospital care characteristics

**Figure 1:**
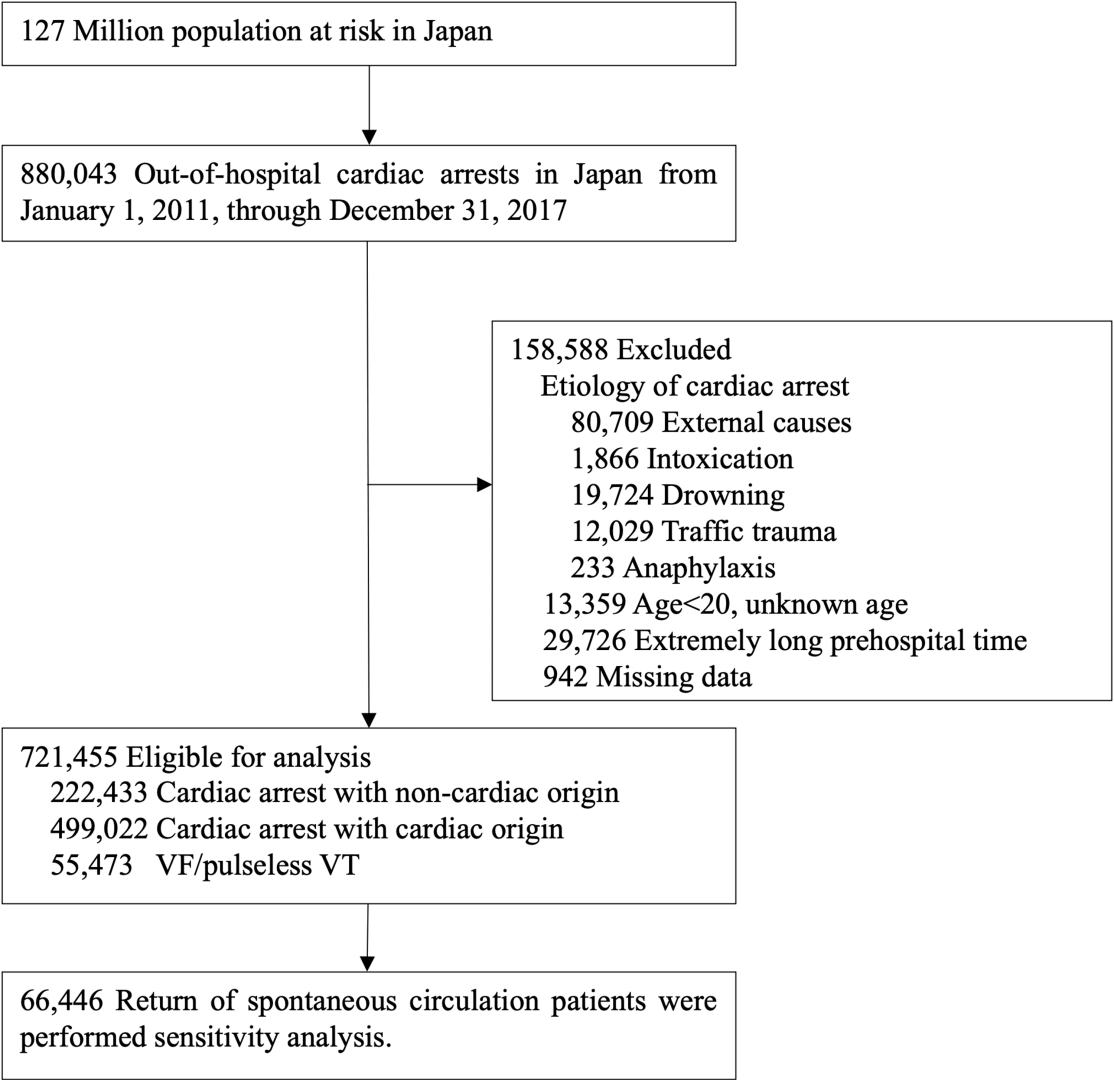
Study participant selection

A total of 880,043 OHCA occurred during the study period in Japan. Of them, 114,561 patients (13.0%) had external causes of cardiac arrest, 942 patients (0.12%) had missing data or were outliers, 13,359 patients (1.52%) with unknown age or whose age was less than 20 years, and 29,726 patients (3.38%) had extremely long prehospital time and were therefore excluded, leaving 721,455 patients (82.0%) eligible for analysis (Figure 1). Patient and prehospital care characteristics for the eligible OHCA patients by geographic region are shown in Table 1.

**Table 1:** Regional, patient, and prehospital characteristics

	North		Northeast		East		Central		Midwest		West		South		Overall	
Characteristics	32,367		64,644		277,199		103,915		107,624		62,732		72,974		721,455	
	4.49%		8.96%		38.42%		14.40%		14.92%		8.70%		10.11%			
Regional characteristics																
Area population	5,381,733		8,982,807		48,233,029		17,251,537		21,512,173		11,283,571		14,449,895		127,094,745	
Population density, residents per square kilometre	68.6		147.9		1468.8		492.5		996.8		239		384.1		340.8	
Acute care hospital no.	12		20		94		46		44		35		33		284	
Acute care hospitals, no. per 1,000,000 population	2.23		2.23		1.95		2.67		2.05		3.10		2.28		2.23	
Hospital and clinic no.	561		593		2347		971		1259		1113		1568		8412	
Patient characteristics																
Age, median (IQR), y	79(68-87)		82(72-88)		80(69-87)		80(71-87)		80(70-87)		81(70-87)		80(69-87)		80(70-87)	
Male sex	18,034(55.7%)		35,760(55.3%)		158,184(57.1%)		58,486(56.3%)		60,229(56.0%)		34,844(55.5%)		40,315(55.2%)		405,852(56.3%)	
Etiology of arrest																
Cardiac	22,010(68.0%)		46,740(72.3%)		196,890(71.0%)		68,688(66.1%)		79,129(73.5%)		41,453(66.1%)		44,112(60.4%)		499,022(69.2%)	
No-cardiac	10,357(32.0%)		17,904(27.7%)		80,309(29.0%)		35,227(33.9%)		28,495(26.5%)		21,279(33.9%)		28,862(39.6%)		222,433(30.8%)	
VF or pulseless VT as initial cardiac rhythm	2,736(8.5%)		4,633(7.2%)		22,167(8.0%)		7,819(7.5%)		8,072(7.5%)		4,205(6.7%)		5,841(8.0%)		55,473(7.7%)	
Prehospital characteristics																
Type of bystander-witness status																
No witness	19,704(60.9%)		37,419(57.9%)		158,062(57.0%)		59,453(57.2%)		61,083(55.1%)		36,868(58.8%)		42,687(58.4%)		415,276(57.3%)	
Layperson	9,880(30.5%)		21,957(34.0%)		96,218(34.7%)		35,804(34.5%)		40,620(36.7%)		20,347(32.4%)		24,594(33.7%)		249,420(34.4%)	
Healthcare providers	2,791(8.6%)		5,279(8.2%)		22,925(8.3%)		8,666(8.3%)		9,095(8.2%)		5,530(8.8%)		5,793(7.9%)		60,079(8.3%)	
CPR by bystander																
No bystander CPR	18,415(57.0%)		29,744(46.4%)		159,160(57.5%)		47,685(46.0%)		57,074(53.2%)		32,850(52.5%)		32,406(44.4%)		377,334(52.4%)	
Compression-only CPR	12,288(38.0%)		29,969(46.7%)		101,793(36.8%)		49,215(47.4%)		42,832(39.9%)		25,355(40.5%)		32,911(45.1%)		294,363(40.9%)	
Conventional CPR	1,600(5.0%)		4,456(6.9%)		15,618(5.6%)		6,831(6.6%)		7,437(6.9%)		4,366(7.0%)		7,643(10.5%)		47,771(6.6%)	
Use of public-access AED by a bystander	298(0.9%)		661(1.0%)		3,862(1.4%)		1,264(1.2%)		1,126(1.0%)		840(1.3%)		1,014(1.4%)		9,065(1.3%)	
Emergency lifesaving technician present in ambulance	31,774(98.2%)		61,224(94.7%)		273,905(98.8%)		102,103(98.3%)		104,834(97.4%)		61,214(97.6%)		70,666(96.8%)		705,720(97.8%)	
Adrenaline administered	9,597(29.7%)		8,694(13.4%)		55,415(20.0%)		17,686(17.0%)		16,674(15.5%)		10,100(16.1%)		8,796(12.1%)		126,962(17.6%)	
Advanced airway management	21,119(65.2%)		19,245(29.8%)		106,887(38.6%)		51,355(49.4%)		51,807(48.1%)		23,984(38.2%)		18,074(24.8%)		292,471(40.5%)	
Median (IQR) interval, min																
Call to CPR by emergency responder	9(7-12)		10(8-13)		9(7-11)		9(7-11)		9(7-11)		9(7-13)		9(7-12)		9(7-12)	
CPR to hospital arrival	21(16-28)		21(16-29)		24(19-31)		20(16-25)		21(16-26)		21(15-28)		19(14-25)		22(16-28)	
Return of spontaneous circulation	3,419(10.6%)		4,774(7.4%)		26,199(9.5%)		10,658(10.3%)		10,342(9.6%)		4,616(7.4%)		6,438(8.8%)		66,446(9.2%)	

The overall median age was 80 years old (range: 70-87), and male patients were 405,852 patients (56.3%). Ventricular fibrillation or pulseless ventricular tachycardia was the initial rhythm in 55,473 patients (7.7%).

As for prehospital care, bystander CPR occurred in 342,134 patients (47.5%), public AED use was 9,065 patients (1.3%), adrenaline administration was 126,962 patients (17.6%), and advanced airway management was 292,471 patients (40.5%). The time from initial call to initiation of CPR was nine minutes (range: 7-12), the time from initiation of CPR to hospital arrival was 22 minutes (range: 16-28), and 66,446 patients (9.2%) of cases had ROSC in the prehospital setting (range: 7.4-10.6).

Regional variation in incidence and outcomes

The overall unadjusted incidence of OHCA was 81.1 per 100,000 person-years. The adjusted incidence of OHCA per 100,000 population ranged from 84.9 to 111.7 (median: 97.2). The overall patient population with favorable neurological outcomes was 17,685 patients (2.45%). Unadjusted neurological favorable outcomes varied regionally, ranging from 1,058 patients (1.64%) to 2,462 patients (3.37%) (rate difference: 1.73%; 95%CI: 1.57-1.89%). The East and Northeast had significantly lower rates compared to the Midwest region (unadjusted rate ratio: 0.73; 95%CI: 0.70-0.76; p<0.001, 0.63; 95%CI: 0.59-0.67; p<0.001, respectively). The significant difference remained after adjustment for patient factors and prehospital factors (adjusted rate ratio: 0.68; 95%CI: 0.64-0.72; p<0.001, 0.70; 95%CI: 0.67-0.73; p<0.001). The secondary outcome, one-month survival after OHCA, also significantly varied ranging from 2,665 patients (4.12%) to 2,266 patients (7.00%) (rate difference, 2.88%; 95%CI: 2.56-3.20%). The East and Northeast had significantly lower rates compared to the Midwest region (unadjusted rate ratio: 0.62; 95%CI: 0.59-0.65; p<0.001, 0.71; 95%CI: 0.69-0.73; p<0.001, respectively). Table 2 summarizes incidence and survival outcomes by region among all OHCA patients.

**Table 2:** Incidence of al EMS-treated out-of-hospital cardiac arrest and survival outcomes, by geographic region a. Adjusted for age and sex data from the 2015 census for Japan. b. Midwest region including cities of Osaka and Kyoto served as the reference. c. Adjusted for a predefined set of potential confounders, including age, sex, cause of arrest, initiation of a bystander CPR, use of a public-access automated external defibrillator by a bystander, adrenaline administration, advanced airway device placement, presence of an emergency lifesaving technician in an ambulance, time from receipt of a call to CPR by emergency medical service, time from a CPR to hospital arrival, and year.

	North	Northeast	East	Central	Midwest	West	South	Overall
n	32,367	64,644	277,199	103,915	107,624	62,732	72,974	721,455
Area population	5,381,733	8,982,807	48,233,029	17,251,537	21,512,173	11,283,571	14,449,895	127,094,745
Incidence rate per 100,000 person-years								
Unadjusted incidence	85.9	102.8	82.1	86.1	71.5	79.4	72.1	81.1
Adjusted incidence^ a^	97.2	111.7	107.6	107.4	89.5	86.6	84.9	
Neurologically favourable survival, no.	861	1,058	6,283	2,934	2,725	1,362	2,462	17,685
Unadjusted rate, %	2.66	1.64	2.27	2.82	2.53	2.17	3.37	2.45
Unadjusted rate ratio(95%CI) ^b^	0.95(0.89-1.02)	0.63(0.59-0.67)	0.73(0.70-0.76)	0.89(0.85-0.93)	Reference	0.75(0.71-0.80)	1.08(1.02-1.13)	-
Adjusted rate,% ^c^	1.44	0.80	0.83	1.20	1.19	0.96	1.21	
Adjusted rate ratio(95%CI) ^b,c^	1.22(1.14-1.31)	0.68(0.64-0.72)	0.70(0.67-0.73)	1.01(0.96-1.06)	Reference	0.81(0.76-0.86)	1.02(0.97-1.08)	-
1-Month survival, no.	2,266	2,665	13,091	5,861	7,189	3,273	4,934	39,279
Unadjusted rate, %	7.00	4.12	4.72	5.64	6.68	5.22	6.76	5.44
Unadjusted rate ratio(95%CI) ^b^	1.05(1.00-1.10)	0.62(0.59-0.65)	0.71(0.69-0.73)	0.84(0.82-0.87)	Reference	0.78(0.75-0.81)	1.01(0.98-1.05)	-
Adjusted rate,% ^c^	4.47	2.53	2.67	3.37	3.66	3.12	3.68	
Adjusted rate ratio(95%CI) ^b,c^	1.25(1.19-1.31)	0.70(0.67-0.73)	0.73(0.71-0.75)	0.93(0.90-0.96)	Reference	0.86(0.83-0.90)	1.02(0.99-1.06)	-

Sensitivity analyses

Favorable neurological outcomes and one-month survival after OHCA were analyzed in the cardiac and non-cardiac arrest patient groups, as well as in the ventricular fibrillation/tachycardia patient subgroup by region. The results of the sensitivity analysis are summarized in Table 3. Table 4 shows the unadjusted and adjusted outcomes for the OHCA patients who achieved ROSC prior to hospital arrival. The results of both sensitivity analyses were similar to the main analysis; regional variations remained significant. 

**Table 3:** Incidence of EMS-treated out-of-hospital cardiac arrest and survival outcomes by etiology and initial rhythm, by geographic region a. Adjusted for age and sex data from the 2015 census for Japan. b. Midwest region including cities of Osaka and Kyoto served as the reference. c. Adjusted for a predefined set of potential confounders including age, sex, cause of arrest, initiation of a bystander CPR, use of a public-access automated external defibrillator by a bystander, adrenaline administration, advanced airway device placement, presence of an emergency lifesaving technician in an ambulance, time from receipt of a call to CPR by emergency medical service, time from a CPR to hospital arrival, and year.

	North	Northeast	East	Central	Midwest	West	South	Overall
Area population	5,381,733	8,982,807	48,233,029	17,251,537	21,512,173	11,283,571	14,449,895	127,094,745
Non-cardiac origin, no.	10,357	17,904	80,309	35,227	28,495	21,279	28,862	222,433
Incidence rate per 100,000 person-years								
Unadjusted incidence	27.5	28.5	23.8	29.2	18.9	26.9	28.5	25.0
Adjusted incidence ^a^	31.2	31.0	31.1	36.4	23.7	29.5	33.6	
Neurologically favourable survival, no.	198	200	938	531	573	324	649	3,413
Unadjusted rate, %	1.9	1.1	1.2	1.5	2.0	1.5	2.2	1.5
Unadjusted rate ratio(95%CI) ^b^	0.95(0.81-1.12)	0.56(0.47-0.65)	0.58(0.52-0.64)	0.75(0.67-0.84)	Reference	0.76(0.66-0.87)	1.12(1.00-1.25)	
Adjusted rate,% ^c^	1.3	0.6	0.6	0.9	1.1	0.9	1.2	
Adjusted rate ratio(95%CI) ^b,c^	1.25(1.06-1.47)	0.59(0.50-0.70)	0.61(0.55-0.68)	0.88(0.78-0.99)	Reference	0.82(0.72-0.94)	1.17(1.04-1.31)	
1-Month survival, no.	659	670	2,957	1,625	1,886	1,016	1,651	10,464
Unadjusted rate, %	6.4	3.7	3.7	4.6	6.6	4.8	5.7	4.7
Unadjusted rate ratio(95%CI) ^b^	0.96(0.88-1.05)	0.57(0.52-0.62)	0.56(0.53-0.59)	0.70(0.65-0.74)	Reference	0.72(0.67-0.78)	0.86(0.81-0.92)	
Adjusted rate,% ^c^	5.1	2.8	2.8	3.5	4.3	3.6	4.3	
Adjusted rate ratio(95%CI) ^b,c^	1.18(1.07-1.29)	0.64(0.59-0.70)	0.64(0.60-0.68)	0.75(0.68-0.84)	Reference	0.83(0.76-0.89)	1.00(0.93-1.07)	
Cardiac origin, no.	22,010	46,740	196,890	68,688	79,129	41,453	44,112	499,022
Incidence rate per 100,000 person-years								
Unadjusted incidence	58.4	74.3	58.3	56.9	52.5	52.5	43.6	56.1
Adjusted incidence^ a^	66.0	80.5	76.5	71.0	65.8	57.1	50.5	
Neurologically favourable survival, no.	870	1,207	6,024	2,674	3,152	1,314	2,069	17,310
Unadjusted rate, %	4.0	2.6	3.1	3.9	4.0	3.2	4.7	3.5
Unadjusted rate ratio(95%CI) ^b^	0.99(0.92-1.07)	0.65(0.61-0.69)	0.77(0.74-0.80)	0.98(0.93-1.03)	Reference	0.80(0.75-0.85)	1.18(1.11-1.24)	
Adjusted rate,% ^c^	1.6	0.9	0.9	1.4	1.3	1.0	1.3	
Adjusted rate ratio(95%CI) ^b,c^	1.20(1.11-1.29)	0.69(0.64-0.74)	0.71(0.68-0.75)	1.03(0.98-1.09)	Reference	0.80(0.75-0.85)	0.97(0.92-1.03)	
1-Month survival, no.	1,607	1,995	10,134	4,236	5,303	2,257	3,283	28,815
Unadjusted rate, %	7.3	4.3	5.1	6.2	6.7	5.4	7.4	5.8
Unadjusted rate ratio(95%CI) ^b^	1.09(1.03-1.15)	0.64(0.60-0.67)	0.77(0.74-0.79)	0.92(0.88-0.96)	Reference	0.81(0.77-0.85)	1.11(1.06-1.16)	
Adjusted rate,% ^c^	4.1	2.3	2.5	3.2	3.3	2.8	3.3	
Adjusted rate ratio(95%CI) ^b,c^	1.24(1.17-1.31)	0.70(0.66-0.74)	0.75(0.72-0.77)	0.96(0.92-1.00)	Reference	0.85(0.81-0.90)	1.01(0.96-1.05)	
Cardiac origin, initial rhythm VF/VT, no.	2,736	4,633	22,167	7,819	8,072	4,205	5,841	55,473
Incidence rate per 100,000 person-years								
Unadjusted incidence	7.3	7.4	6.6	6.5	5.4	5.3	5.8	6.2
Adjusted incidence ^a^	8.6	8.6	8.2	8.0	6.7	6.3	7.2	
Neurologically favourable survival, no.	576	760	3,555	1,780	1,871	862	1,389	10,793
Unadjusted rate, %	21.1	16.4	16.0	22.8	23.2	20.5	23.8	19.5
Unadjusted rate ratio(95%CI) ^b^	0.91(0.83-1.00)	0.71(0.65-0.77)	0.69(0.65-0.73)	0.98(0.92-1.05)	Reference	0.88(0.82-0.96)	1.03(0.96-1.10)	
Adjusted rate,% ^c^	10.6	6.9	7.2	9.8	9.8	7.7	8.8	
Adjusted rate ratio(95%CI) ^b,c^	1.02(0.93-1.12)	0.71(0.65-0.77)	0.71(0.67-0.75)	1.02(0.95-1.09)	Reference	0.79(0.73-0.86)	0.90(0.84-0.96)	
1-Month survival, no.	937	1,131	5,272	2,511	2,708	1,258	1,909	15,726
Unadjusted rate, %	34.2	24.4	23.8	32.1	33.5	29.9	32.7	28.3
Unadjusted rate ratio(95%CI) ^b^	1.02(0.95-1.10)	0.73(0.68-0.78)	0.71(0.68-0.74)	0.96(0.91-1.01)	Reference	0.89(0.83-0.95)	0.97(0.92-1.03)	
Adjusted rate,% ^c^	21.5	13.5	14.1	17.9	18.2	15.3	16.7	
Adjusted rate ratio(95%CI) ^b,c^	1.11(1.03-1.20)	0.75(0.70-0.80)	0.75(0.71-0.78)	0.99(0.94-1.05)	Reference	0.85(0.79-0.91)	0.91(0.85-0.96)	

**Table 4:** Unadjusted and adjusted outcomes of EMS-treated out-of-hospital cardiac arrest with return of spontaneous circulation, by geographic region a. Midwest region including the cities of Osaka and Kyoto served as the reference. b. Adjusted for a predefined set of potential confounders including age, sex, cause of arrest, initiation of a bystander CPR, use of a public-access automated external defibrillator by a bystander, adrenaline administration, advanced airway device placement, presence of an emergency lifesaving technician in an ambulance, time from receipt of a call to CPR by emergency medical service, time from a CPR to hospital arrival, and year.

	North	Northeast	East	Central	Midwest	West	South	Overall
Area population	3,419	4,774	26,199	10,658	10,342	4,616	6,438	66,446
Neurologically favourable survival, no.	826	1,139	5,826	2,595	2,842	1,224	2,027	16,479
Unadjusted rate, %	24.2	23.9	22.2	24.3	27.5	26.5	31.5	24.8
Unadjusted rate ratio (95%CI)^a^	0.88(0.81-0.95)	0.87(0.81-0.93)	0.81(0.77-0.85)	0.89(0.84-0.93)	Reference	0.96(0.90-1.03)	1.15(1.08-1.21)	
Adjusted rate,%^ b^	16.0	12.8	12.7	14.5	15.5	14.3	15.4	
Adjusted rate ratio (95%CI)^b^	1.02(0.90-1.16)	0.81(0.71-0.92)	0.83(0.77-0.92)	0.94(0.86-1.03)	Reference	0.91(0.81-1.03)	1.02(0.91-1.13)	
1-Month survival, no.	1,192	1,373	8,342	3,699	3,339	1,570	2,686	22,201
Unadjusted rate, %	34.9	28.8	31.8	34.7	32.3	34.0	41.7	33.4
Unadjusted rate ratio (95%CI)^a^	1.03(0.97-1.10)	0.90(0.84-0.96)	0.84(0.81-0.88)	0.91(0.87-0.95)	Reference	1.00(0.95-1.07)	1.11(1.06-1.17)	
Adjusted rate,% ^b^	38.3	30.2	29.8	32.1	35.4	33.5	34.1	
Adjusted rate ratio (95%CI) ^b^	1.03(0.95-1.11)	0.83(0.77-0.88)	0.82(0.78-0.86)	0.94(0.89-0.99)	Reference	0.92(0.86-0.98)	0.99(0.93-1.05)	

## Discussion

In this large OHCA registry in Japan, we found significant regional differences in favorable neurological outcomes and one-month survival after OHCA, which persisted even after adjustment for both patient and prehospital factors, as well as in the subgroup analysis for the patients who achieved ROSC in the field. In addition, favorable neurological outcomes and not 1-month survival rates after OHCA have also increased overall [9]. 

The incidence and outcomes of OHCA have been reported to vary widely worldwide, with Asia having a lower survival to discharge rate than other continents [21,22]. This variability is not only present across continents but also within each country. A United States-based study utilizing the Cardiac Arrest Registry to Enhance Survival showed significant differences in survival to discharge and survival with functional recovery across 132 US counties; survival to discharge ranged from 3.4% to 22.0% (median odds ratio: 1.40; 95%CI: 1.32-1.46) and survival with favorable neurological outcome ranged from 0.8% to 21.0% (median odds ratio: 1.53; 95%CI: 1.43-1.62) [5]. These differences were partially explained by regional variation in patient sociodemographics, cardiac arrest characteristics, and, most importantly, rate of bystander CPR, which appeared to have a particularly large impact [5]. A multicenter registry of 28 European countries similarly showed variations in OHCA prognosis between countries [6]. One-third of the study’s patients overall achieved ROSC in the field, with rates ranging from 8% to 42%. Survival to hospital discharge occurred in 8% of patients overall, ranging from 0% to 18%. Higher rates were present in patients who received CPR by bystanders, especially full CPR (with ventilation), rather than compression-only CPR or no bystander CPR at all [6].

**Figure 2:**
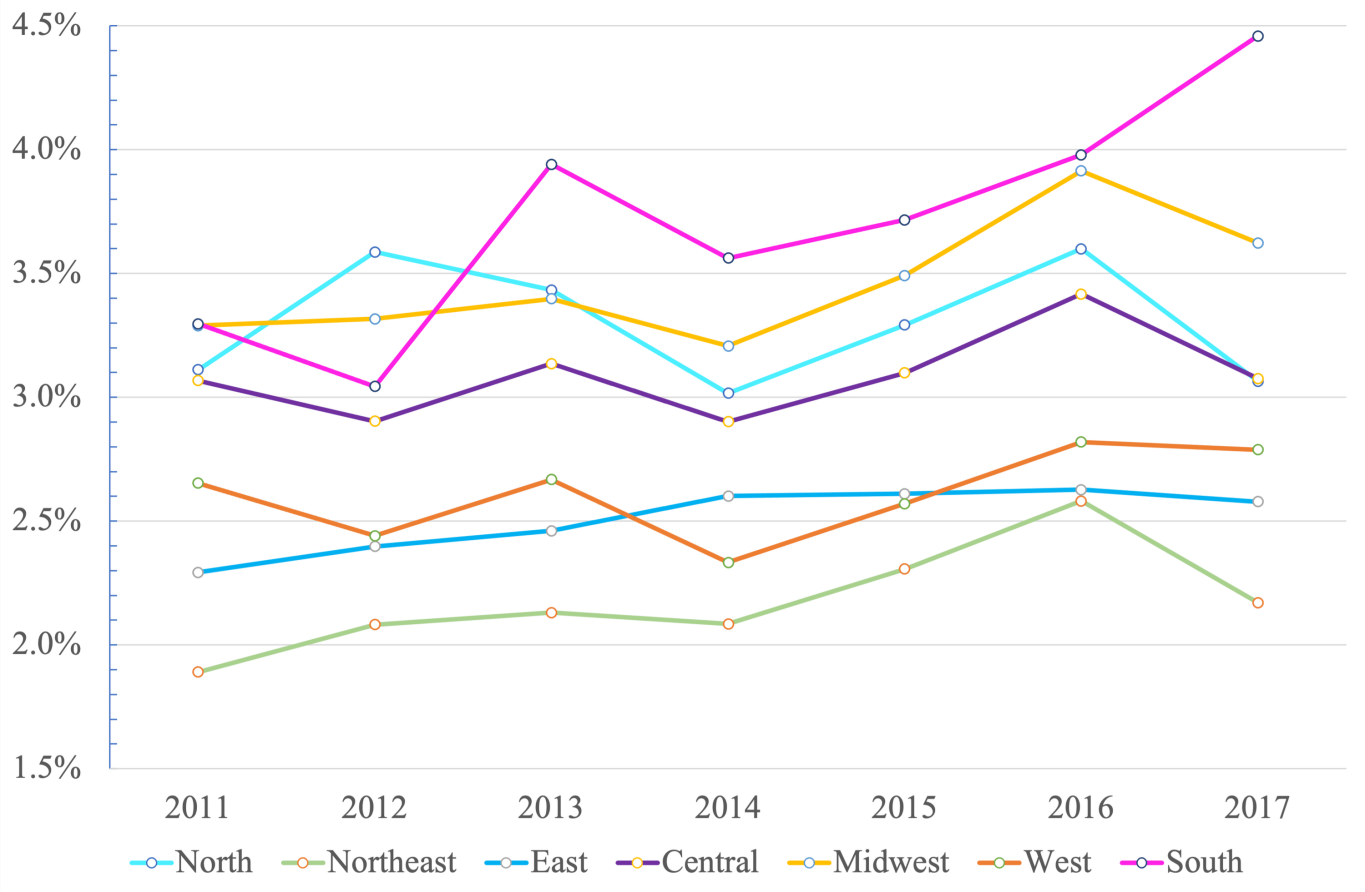
Regional incidence of favorable neurological outcome patients

This regional variability in favorable neurological outcomes and one-month survival after OHCA was present in our study as well. When compared to previous data, these regional differences seem to have narrowed (Figure 2). In our results, the adjusted OR was 0.68 (95%C: 0.64-0.72). A previous study by Hasegawa et al. [9] reported a two-fold regional difference in neurologically favorable survival after OHCA between 2005 and 2010 (adjusted rate ratio: 0.52; 95%CI: 0.51-0.54) [9]. Longitudinal analysis in the future is awaited.

Several possible explanations for this variability exist. The difference in median age amongst regions was proposed, but the variability persisted after adjusting for age. The different population densities between urban and rural areas may be another explanation. However, the population density and the number of acute care hospitals per population do not seem to vary significantly between regions (Table 1) and therefore would not explain the variability in outcomes. Factors such as the patient's economic status and underlying health conditions could affect the outcome. The level of economic development in each region is shown in the supplement as Gross Domestic Product (Supplement Appendix C). Variability in prehospital care is an unlikely contributor to this regional variability; the initial treatment by EMS is standardized by the Japan Resuscitation Council guidelines, which were established in reference to ILCOR [16] and are updated every five years [23]. Regional differences persisted despite adjusting for multiple factors in prehospital care, including rates of bystander CPR and EMS response times, which have been postulated as possible causes of this variability in other countries [5-7]. In contrast, hospital care is difficult to standardize completely. For example, differences in post-arrest care between hospitals in therapeutic temperature management, percutaneous coronary intervention, and hospital multidisciplinary care may contribute to our findings. The variations in survival outcomes emphasize the importance of optimizing multiple components of the regional treatment system, not only out-of-hospital but also in-hospital post-resuscitation care, in order to improve outcomes after cardiac arrest [24]. The observed regional differences in the incidence of patients with good neurologic outcomes are not small values considering the large public health burden of OHCA. If the good neurological outcome rate improved from 1.64% to 3.37% throughout Japan, a difference of one person with favorable neurological outcome per approximately 57.8 resuscitation actions would occur. This would lead to a total of approximately 20,119 patients with good neurological outcomes during the study period. This translates to the avoidance of many deaths and disabilities due to OHCA if the current regional differences are eliminated and best practices are provided.

It is worth noting that the survival rates reported in our study are much lower than those reported from other countries (such as the United States and Europe) [5,6,21] and are in line with prior studies from Asia [9,21]. The advanced age of our study population is a possible factor; the average age of OHCA patients in the United States is 63.7 years [5] and in Europe is 67.6 years [6], whereas in this study the average age is 80 years.

In addition, the etiology of OHCA in the Japanese population may be different than in other countries. For example, up to 16% of nontraumatic OHCA in Japan may be due to subarachnoid hemorrhage [25], negatively affecting the rates of ROSC and favorable neurological outcomes. According to the systematic review and meta-analysis on the prevalence of ICH in non-traumatic OHCA, it was reported that subarachnoid hemorrhage was the cause of OHCA is 4.15% [26]. Another important reason for the low survival rates overall is EMS's severely limited ability to terminate resuscitation in the field, which occurs in up to 30% of OHCA in some international studies [6]. In addition, advanced care planning is not widely used in Japan [27]; some patients who do not wish to be resuscitated are being transported to hospitals and resuscitated [28,29].

Finally, the rates of reported bystander CPR in Japan are low; only 47.5% of our study population received CPR from bystanders. According to a study that observed the status of OHCA in various countries, there were large differences in bystander CPR depending on the country, with bystander CPR being performed in more than 70% of cases in Denmark, Norway, Australia, New Zealand, and Ireland [30]. In contrast, in countries such as the United States, Japan, Germany, and Italy, it was around 50% [30]. This highlights an important area of potential improvement from a public health standpoint.

The strengths of this study, firstly, are that it is a comprehensive database of OHCA patients throughout Japan and that it is recorded without omission by emergency services teams nationwide. Additionally, since it is almost rare for emergency services teams not to transport patients to hospitals, almost all out-of-hospital cardiac arrest patients are registered.

Our study had several limitations. First, we used only prehospital patient data and did not include post-resuscitation data that might have affected outcomes. Second, patient factors such as underlying health conditions (e.g., hypertension, diabetes), dietary habits, and activities of daily living were not recorded. Therefore, regional differences in cardiovascular risks are unknown. As with all epidemiological studies, data integrity, validity, ascertainment bias, and misclassifications were potential limitations. In this analysis, 942 patients (0.11%) were excluded due to obvious outliers or missing data. Finally, despite adjusting for potential covariates, it is not possible to exclude other possible residual confounding factors.

## Conclusions

In this post-hoc analysis, a nationwide, population-based study in Japan, we found that regional differences in favorable neurologic outcomes of OHCA patients persist. Although the incidence of patients with a favorable neurological outcome is increasing, the fact that regional differences still exist is a major public health problem. It is prudent to further investigate the causes of this difference as a first step to improving the outcomes of OHCA in the future.

## Figures and Tables

**Figure 3:**
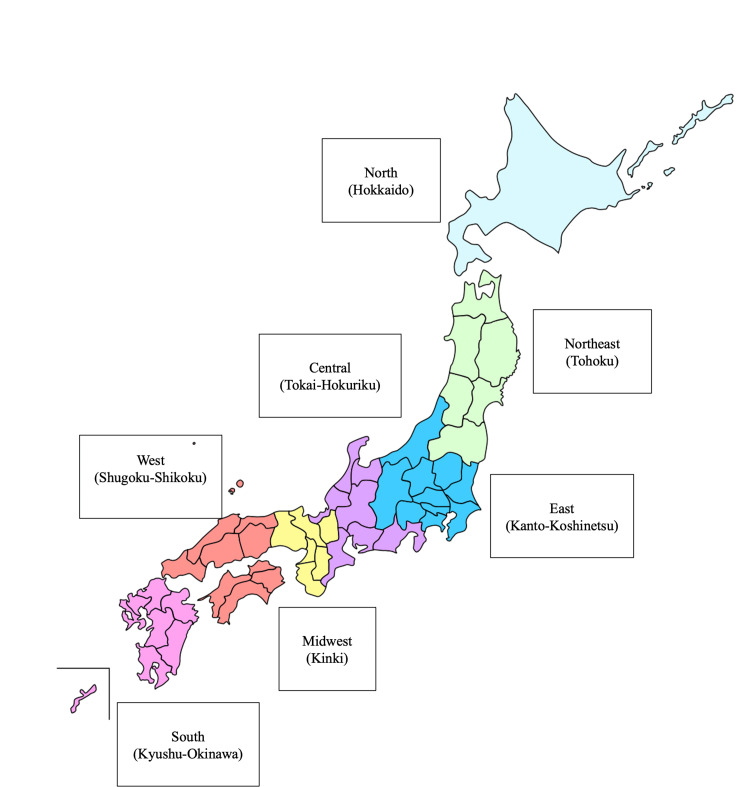
Japanese regions according to the Ministry of Health, Labour, and Welfare

## Appendices

Supplemental Appendix A. Prefectures in Japanese Regions according to the Ministry of Health, Labour, and Welfare.

**Table 5:** Prefectures in Japanese regions according to the Ministry of Health, Labour, and Welfare

Prefectures in Japanese Regions according to Ministry of Health, Labour, and Welfare							
North (Hokkaido)	Northeast (Tohoku)	East (Kanto-Koshinetsu)	Central (Tokai-Hokuriku)	Midwest (Kinki)	West (Chugoku-Shikoku)	South (Kyushu-Okinawa)	
Hokkaido	Fukushima	Tokyo	Toyama	Kyoto	Hiroshima	Fukuoka	
	Aomori	Ibaraki	Ishikawa	Osaka	Tottori	Saga	
	Iwate	Tochigi	Gifu	Fukui	Shimane	Nagasaki	
	Miyagi	Gunma	Shizuoka	Shiga	Okayama	Kumamoto	
	Akita	Saitama	Aichi	Hyogo	Yamaguchi	Oita	
	Yamagata	Chiba	Mie	Nara	Tokushima	Miyazaki	
		Kanagawa		Wakayama	Kagawa	Kagoshima	
		Nigata			Ehime	Okinawa	
		Yamanashi			Kochi		
		Nagano					

Supplementary Appendix B. Japanese Regions according to the Ministry of Health, Labour, and Welfare.

Supplementary Appendix C. GDP for each region in Japan.

**Table 6:** GDP for each region in Japan Citation from prefectural economic accounts (2015): Cabinet Office, Government of Japan.

Region	GDP (million yen)
North	20,540,923
Northeast	34,488,192
East	247,674,181
Central	87,875,389
Midwest	89,127,762
West	45,284,696
South	52,360,145
